# Increased Virulence in Sunflower Broomrape (*Orobanche cumana* Wallr.) Populations from Southern Spain Is Associated with Greater Genetic Diversity

**DOI:** 10.3389/fpls.2016.00589

**Published:** 2016-05-03

**Authors:** Alberto Martín-Sanz, Jebri Malek, José M. Fernández-Martínez, Begoña Pérez-Vich, Leonardo Velasco

**Affiliations:** ^1^Pioneer Hi-Bred Agro Servicios Spain SLSevilla, Spain; ^2^Institute for Sustainable Agriculture, Consejo Superior de Investigaciones CientíficasCórdoba, Spain

**Keywords:** sunflower broomrape, *Orobanche cumana*, new race, virulence, genetic diversity, gene pools, genetic recombination

## Abstract

*Orobanche cumana* Wallr. (sunflower broomrape) is a holoparasitic weed that infects roots of sunflower in large areas of Europe and Asia. Two distant *O. cumana* gene pools have been identified in Spain, one in Cuenca province in the Center and another one in the Guadalquivir Valley in the South. Race F has been hypothesized to have arisen by separate mutational events in both gene pools. In the Guadalquivir Valley, race F spread in the middle 1990’s to become predominant and contained so far with race F hybrids. Recently, enhanced virulent populations of *O. cumana* have been observed in commercial fields parasitizing race F resistant hybrids. From them, we collected four independent populations and conducted virulence and SSR marker-based genetic diversity analysis. Virulence essays confirmed that the four populations studied can parasitize most of the race F resistant hybrids tested, but they cannot parasitize the differential inbred lines DEB-2, carrying resistance to race F and G, and P-96, resistant to F but susceptible to races G from other countries. Accordingly, the new populations have been classified as race G_GV_ to distinguish them from other races G. Cluster analysis with a set of populations from the two Spanish gene pools and from other areas, mainly Eastern Europe, confirmed that race G_GV_ populations maintain close genetic relatedness with the Guadalquivir Valley gene pool. This suggested that increased virulence was not caused by new introductions from other countries. Genetic diversity parameters revealed that the four populations had much greater genetic diversity than conventional populations of the same area, containing only alleles present in the Guadalquivir Valley and Cuenca gene pools. The results suggested that increased virulence may have resulted from admixture of populations from the Guadalquivir Valley and Cuenca followed by recombination of avirulence genes.

## Introduction

*Orobanche cumana* Wallr. (sunflower broomrape) is a holoparasitic plant species with a restricted range of hosts both in the wild, where it mainly parasitizes *Artemisia* spp., as well as in agricultural fields, where it only grows on sunflower (Fernández-Martínez et al., 2015). The parasitic interaction between sunflower and *O. cumana* generally follows a gene for gene model, with resistance in sunflower (Vrânceanu et al., 1980) and avirulence in *O. cumana* (Rodríguez-Ojeda et al., 2013b) controlled by dominant alleles at single loci. Nonetheless, more complex genetic control of resistance to *O. cumana* has been also reported in some sunflower resistant sources, including two dominant genes (Domínguez, 1996), one dominant and one recessive gene (Akhtouch et al., 2002; Akhtouch et al., 2016), one dominant and one modifying gene (Velasco et al., 2007), one recessive gene (Imerovski et al., 2016), two recessive genes (Rodríguez-Ojeda et al., 2001; Akhtouch et al., 2002), or polygenic genetic control (Labrousse et al., 2004).

The general occurrence of a gene for gene interaction between sunflower and *O. cumana* and the associated development of physiological races of the parasite is an exception in parasitic systems involving *Orobanche* spp., which are in general under quantitative or horizontal genetic control (Pérez-Vich et al., 2013). Vrânceanu et al. (1980) reported the existence of five races of *O. cumana* named as A to E, controlled by resistance genes *Or1* to *Or5*. New populations overcoming *Or5* resistance and named as race F were identified from the middle 1990’s in most of the areas infested by *O. cumana*, namely Spain (Alonso et al., 1996), Romania (Pacureanu-Joita et al., 2004), Turkey (Kaya et al., 2004), Bulgaria (Shindrova, 2006), Ukraine (Burlov and Burlov, 2010), and Russia (Antonova et al., 2013). Nowadays, increasingly virulent populations classified as races G and H are becoming predominant in countries around the Black Sea (Kaya, 2014).

*O. cumana* has been traditionally considered as one of the few exceptions of the genus that are self-pollinating, which has been based on its flower morphology, with small lower lips that do not facilitate the action of big pollinators such as bees and bumblebees (Satovic et al., 2009), and the structure of its populations, characterized by low intra-population and large inter-population genetic variation (Gagne et al., 1998). However, experimental research using a mutant line lacking anthocyanin pigmentation evidenced the existence of a percentage of cross fertilization in this species of up to 40% under the conditions of the experiments, in which small insects were identified as pollinating agents (Rodríguez-Ojeda et al., 2013a).

*Orobanche cumana* is not present in the wild in Spain, but exclusively found in agricultural fields parasitizing sunflower (Pujadas-Salvà and Velasco, 2000). Recent studies have identified two well separated gene pools, one in Cuenca province in Central Spain and another one in the Guadalquivir Valley in southern Spain (Pineda-Martos et al., 2013; Molinero-Ruiz et al., 2014). The study of Pineda-Martos et al. (2013), conducted on 50 populations collected from both areas of Spain, reported very low intra-population and inter-population genetic diversity within each gene pool, which was hypothesized to be caused by a founder effect in separate introductions. Even so, greater genetic diversity was detected in a small number of populations, in which the presence of individuals from both gene pools and heterozygotes resulting from their hybridization were identified. Interestingly, both gene pools included populations classified as race E and race F that showed very high genetic similarity, suggesting that race F probably arose from punctual mutations within each gene pool (Pineda-Martos et al., 2013). To avoid misunderstanding, we are naming race F from the Guadalquivir Valley as F_GV_.

The Guadalquivir Valley in southern Spain is one of the main areas of sunflower cultivation in this country. *O. cumana* race F_GV_ appeared in this area in the middle 1990’s and spread rapidly to become predominant until the present day. In 2014, small spots of *O. cumana* plants parasitizing sunflower hybrids resistant to race F_GV_ were observed in several fields. The objectives of this research were (i) to evaluate the virulence of these populations on a set of differential lines and hybrids; (ii) to analyze their genetic relatedness to local and foreign populations in order to test whether they resulted from new introductions; and (iii) to determine the genetic diversity of the populations.

## Materials and Methods

### *Orobanche cumana* Populations

Spots of *O. cumana* plants growing on sunflower hybrids with complete resistance to race F_GV_ were identified in 2014 in Sevilla Province, in the Guadalquivir Valley. From them, seeds of mature *O. cumana* plants were collected in four independent sunflower fields at three distant locations in July 2014. Populations BR-24 (36° 58′ 47.9′′ N; 5°54′ 57.1′′ W) and BR-27 (36° 59′ 34.0′′ N; 5° 54′ 49.2′′ W) were collected in Las Cabezas de San Juan, population BR-25 (37° 27′ 59.4′′ N; 5° 40′ 24.2′′ W) was collected in Carmona, and population BR-28 (37° 24′ 25.8′′ N; 5° 14′ 47.6′′ W) was collected in Écija, in the four cases on commercial sunflower hybrids resistant to race F_GV_. The reason for not informing on the names of the hybrids on which the populations were collected is given below. *O. cumana* plants were found scattered in patches of no more than 15 m of diameter. The longest distance between the fields where the populations were collected was 98 km between BR-24 and BR-28, while the shortest distance was 1,6 km between BR-24 and BR-27. Seeds of a conventional race F population of the Guadalquivir Valley (F_GV_) named as SP were used as a control in the phenotypic evaluations. Seeds of SP population were collected in 2001 in Écija (Sevilla) and the population is multiplied periodically on cultivar NR5, resistant to races A to E and susceptible to race F_GV_. Six *O. cumana* populations from several Eastern European countries, all of them classified as race G on the basis of their ability to parasitize race F resistant hybrids, were used for comparative phenotypic evaluation of the alleged new race G population from the Guadalquivir Valley. They were collected in Ialomita County, Romania (G_RO_), Thrace region of Greece (G_GR_), Edirne area of Turkey (G_TK_), southern area of Bulgaria (G_BU_), Rostov area of Russia (G_RU_), and Lugansk area of Ukraine (G_UK_).

Twenty *O. cumana* populations from Spain and other countries were used as a reference to compare the molecular genetic similarity between the four populations under study with those from other geographical areas. The selection of the populations did not pretend to be an exhaustive representation of the main sunflower production areas, but just a set of geographically diverse populations to test the hypothesis on whether the new populations with increased virulence evolved in the Guadalquivir Valley area or, contrarily, whether they might have resulted from new introductions. The set of reference populations included four populations from the Guadalquivir Valley: CO-02, SE-10 (Pineda-Martos et al., 2013), EK-23 (Rodríguez-Ojeda et al., 2013b), and SP; four populations from Cuenca province in Central Spain: IASCum-4, CU-05, CU-07, CU-12 (Pineda-Martos et al., 2014b); one population from Serbia (Boro-9); one population from Romania (Boro-10); one population from Israel (Boro-11); two populations from Turkey (Boro-14, Boro-15); and seven populations from Bulgaria (Boro-16 to Boro-22). In all cases seeds were collected in the respective areas and tissue from 20 individual plants growing on sunflower susceptible line B117 was collected for DNA extraction. Equal amounts of DNA of the plants from each population were pooled and used as a template for PCR amplification.

The study of intrapopulation diversity was based on the four populations under study (BR-24, BR-25, BR-27, BR-28). Two conventional populations of the Guadalquivir Valley (SE-10) and Cuenca (IASCum-4) were used as a reference for both gene pools based on a previous study (Pineda-Martos et al., 2013). DNA from 12 randomly selected individual plants per population was used as a template for PCR amplification.

### Sunflower Lines and Hybrids

Four differential inbred lines, two commercial hybrids susceptible to race F_GV_, and 13 commercial hybrids with complete resistance to race F_GV_ were used in the experiments conducted to evaluate the virulence of the *O. cumana* populations. The inbred lines were B117, susceptible to all tested *O. cumana* races; NR5, resistant to Guadalquivir Valley’s race E; P-96, resistant to race F_GV_ and susceptible to races G from other countries; and DEB-2, resistant to both race F_GV_ and races G. Commercial hybrids susceptible to race F_GV_ were 63D82 and P64LE19 (Pioneer). Commercial hybrids resistant to race F_GV_ were named as Hybrid 1 through Hybrid 13. All of them are hybrids widely cultivated in the Guadalquivir Valley area for several years with complete resistance to race F_GV_. The names of the hybrids are not provided because the objective of the research was not to conduct a survey on resistance of commercial hybrids to a new race, but just to ensure that the new populations under study can be considered as a new race. Accordingly, the hybrids were not selected following the criterion of representativeness, and some seed companies may be overrepresented and others not included in the study, which might result in an unbalanced impact on the commercial interests of seed companies.

### Phenotypic Evaluation

Virulence of the four populations BR-24, BR-25, BR-27, and BR-28 together with SP, G_RO_, G_GR_, G_TK_, G_BU_, G_RU_, and G_UK_ used as a control were evaluated on four differential lines (B117, NR5, P-96, DEB-2) and six hybrids (63D82, P64LE19, Hybrid 1, Hybrid 2, Hybrid 3, Hybrid 4). Because of the limitation in the amount of *O. cumana* seed available, the experiments were conducted on multi-pot trays with a pot volume of 40 cm^3^. Twenty plants of each line or hybrid, separated into two replications of ten plants each, were evaluated for each *O. cumana* population. An additional experiment based on a single replication of 10 plants was conducted to test the virulence of population BR-28, for which additional seeds were available, on 14 hybrids: 13 commercial hybrids resistant to race F_GV_ and the control 63D82, susceptible to race F_GV_.

In all experiments, the soil consisted in a mixture of sand and peat (1:1 by vol). The soil was inoculated with *O. cumana* seeds at an approximate concentration of 0.28 mg of seeds per g of soil. This roughly corresponds to 185 seeds per g of soil or 4,000 seeds per pot. The soil mixture containing the *O. cumana* seeds was carefully mixed to obtain a homogeneously infested substrate. Sunflower seeds were germinated in moistened filter paper and then planted in the pots. The plants were grown in a growth chamber at 25/20°C (day/night) with a 16-h photoperiod, and photon flux density of 300 μmol m^-2^ s^-1^.

Phenotypic evaluation of virulence was conducted by determining the level of *O. cumana* incidence (percentage of sunflower plants sustaining the growth of *O. cumana* shoots), number of *O. cumana* attachments per sunflower plant, and the *O. cumana* stage of development classified as: T1 = small nodules with diameter less than 2 mm; T2 = nodules with diameter greater than 2 mm and stem differentiation not yet apparent; T3 = nodules typically between 5 and 10 mm in which stem differentiation can be clearly observed or even the stem has grown several centimeters. In this paper we are only reporting the sum of nodules at T2 and T3 stages.

### Tissue Collection, DNA Extraction, and SSR Analysis

Individual *O. cumana* plants showing well developed stems (T3 stage) were collected on sunflower line B117 for each population. The plants were frozen at -80°C, lyophilized and ground to a fine powder. DNA was extracted from individual plants using a modified version of the protocol described in Pérez-Vich et al. (2004). For the study of interpopulation diversity, equal amounts of DNA of 24 *O. cumana* plants from each population were pooled and used as a template for PCR amplification. For the study of intrapopulation diversity, DNA from individual plants was used as a template for PCR amplification. Microsatellite analyses were carried out as described in Pineda-Martos et al. (2014b) using 20 high-quality, polymorphic SSR primer pairs reported in that work: Ocum-003, Ocum-023, Ocum-052, Ocum-059, Ocum-063, Ocum-070, Ocum-074, Ocum-075, Ocum-081, Ocum-087, Ocum-091, Ocum-092, Ocum-108, Ocum-122, Ocum-141, Ocum-151, Ocum-160, Ocum-174, Ocum-196, and Ocum-197. Amplification products were analyzed by gel electrophoresis using 3% Metaphor agarose (BMA, Rockland, ME, USA) in 1x TBE buffer and SaveView Nucleic Acid Stain (NBS Biologicals Ltd., Huntingdon, UK) and visualized under UV light. A 100 bp DNA ladder (Solis BioDyne, Tartu, Estonia) was used as a standard molecular weight marker to get an approximate size of DNA fragments. Bands were scored manually with the aid of Quantity One^®^ 1-D Analysis Software (Bio-Rad Laboratories Inc., Hercules, CA, USA). Amplified fragments were scored for the presence (1) or absence (0) of homologous bands and compiled into a binary data matrix or for their estimated molecular weight and compiled into a codominant data matrix.

### Analysis of Inter-population Similarities

A dissimilarity matrix was created using DICE dissimilarity index, from which cluster analysis was conducted using the UPGMA (Unweighted Pair Group Method with Arithmetic Mean) method of NTSYSpc ver. 2.21q (Applied Biostatistics, Inc, Port Jefferson, NY, USA). The cophenetic correlation coefficient was calculated to test the goodness of fit of the dendrogram to the original dissimilarity matrix.

### Genetic Diversity Analysis

The percentage of polymorphic loci (P), the average observed number of alleles (Na), the number of different alleles with a frequency ≥5% (Na ≥5%), the number of effective alleles (Ne), the number of private alleles unique to a single population (Npa), the observed and expected heterozygosity (Ho and He), the Shannon’s diversity index (I), and the fixation index (Fis) were calculated for all loci at each population using GenAlEx ver. 6.5. Pairwise genetic distances between populations were calculated as the genetic distance coefficient GST using GenAlEx ver. 6.5 using 1000 random permutations to assess significance. The matrix of GST pairwise distances was used as input for a principal coordinates analysis (PCoA).

The average number of pairwise differences between individuals within each population was calculated as a measure of intrapopulation genetic diversity. Analysis of molecular variance (AMOVA) was conducted on the distance matrix to separate total variance into variance attributable to differences between individuals within a population and variance attributable to differences between populations. In both cases, Arlequin ver. 3.5.2.2 (L. Excoffier, CMPG, University of Bern, Switzerland) was used.

## Results

### Phenotypic Evaluation of Virulence

The four alleged race G *O. cumana* populations BR-24, BR-25, BR-27, and BR-28 showed a similar pattern of virulence, resulting in 100% of parasitized plants and a high number of attachments per plant (9.2 to 25.8) on the race F_GV_ susceptible genotypes B117, 63D82, NR5, and P64LE19, high level of incidence (75 to 100%) and moderate number of attachments per plant (3.2 to 10.1) on Hybrid 1 and Hybrid 3, moderate level of incidence (20 to 50%) and low number of attachments per plant (1.3 to 2.0) on Hybrid 2, low level of incidence (0 to 15%) and low number of attachments per plant (1.0 to 2.0) on Hybrid 4, and no parasitization on lines P96 and DEB2 (**Table 1**). Population SP, a conventional race F_GV_ population used as a control, only parasitized on the four race F susceptible genotypes, with 100% incidence and between 10.8 and 22.2 attachments per plant, similar to the populations under study (**Table 1**). Race G populations from Romania (G_RO_), Greece (G_GR_), Turkey (G_TK_), Bulgaria (G_BU_), Russia (G_RU_), and Ukraine (G_UK_) parasitized in all cases line P96, with incidence between 50% (G_RO_ and G_BU_) and 100% (G_GR_ and G_TK_), which marked a significant difference between the four populations from the Guadalquivir Valley and race G populations from Eastern Europe. None of the race G populations parasitized on DEB2 line. The six race G populations from Eastern Europe showed some differences between them, from G_BU_ that failed to parasitize the four race F resistant hybrids to G_RO_, G_TK_, and G_UK_ that parasitized all of them (**Table 1**).

**Table 1:** Percentage of susceptible plants (%S) and average number of *Orobanche cumana* attachments in susceptible plants (Xs) in ten sunflower lines and hybrids evaluated with *O. cumana* populations BR-24, BR-25, BR-27, BR-28, and SP (race F_GV_) from the Guadalquivir Valley, and populations classified as race G from Romania (G_RO_), Greece (G_GR_), Turkey (G_TK_), Bulgaria (G_BU_), Russia (G_RU_), and Ukraine (G_UK_).

	*Orobanche cumana* populations																					
	*BR-24*		*BR-25*		*BR-27*		*BR-28*		*SP*		*G_RO_*		*G_GR_*		*G_TK_*		*G_BU_*		G_RU_		G_UK_	
Line/Hybrid^a^	%S	X_S_	%S	X_S_	%S	X_S_	%S	X_S_	%S	X_S_	%S	X_S_	%S	X_S_	%S	X_S_	%S	X_S_	%S	X_S_	%S	X_S_
B117	100	25,8	100	12,7	100	20,3	100	11,3	100	22,2	100	5,1	100	8,5	100	14,0	100	10,5	100	9,2	100	11,0
63D82	100	14,8	100	10,1	100	13,1	100	8,0	100	11,8	100	5,1	100	12,4	100	12,2	100	14,0	100	15,0	100	10,5
NR5	100	16,8	100	15,2	100	9,2	100	7,7	100	10,8	100	5,7	100	12,2	100	14,5	100	16,4	100	14,7	100	11,8
P64LE19	100	15,8	100	11,3	100	13,3	100	7,6	100	15,7	100	8,0	100	11,2	100	14,8	100	14,8	100	15,0	100	15,0
P96	0	0,0	0	0,0	0	0,0	0	0,0	0	0,0	50	1,4	100	4,8	100	7,8	50,0	4,0	90	3,0	90	4,4
DEB2	0	0,0	0	0,0	0	0,0	0	0,0	0	0,0	0	0,0	0	0,0	0	0,0	0	0,0	0	0,0	0	0,0
Hybrid 1	95	7,8	85	5,4	100	10,1	75	5,7	0	0,0	100	3,2	100	2,4	100	7,3	0,0	0,0	90	1,8	100	3,3
Hybrid 2	30	1,5	20	2,0	50	1,3	30	1,3	0	0,0	80	2,0	0	0,0	80	2,0	0,0	0,0	40	1,5	30	1,0
Hybrid 3	85	4,4	75	3,5	100	6,2	90	3,2	0	0,0	100	2,8	40	2,5	100	11,6	0,0	0,0	70	3,2	100	3,7
Hybrid 4	5	2,0	0	0,0	10	2,0	15	1,0	0	0,0	30	1,0	0	0,0	30	1,6	0,0	0,0	0	0,0	70	1,0

*The number of plants evaluated per line/hybrid and population was 20 in all cases.*

*^a^Previous information indicated that B117 and 63D82 are susceptible to all O. cumana races, NR5 and P64LE19 are resistant to race E, P96 is resistant to races E and F_GV_, DEB2 is resistant to races E, F_GV_, and G_TK_, and Hybrids 1 to 4 are resistant to races E and F_GV_.*

A second experiment, focused on the evaluation of population BR-28 with a set of 12 commercial hybrids resistant to race F_GV_, revealed a degree of incidence between 56 and 100% and average number of attachments per plant between 1.4 and 12.8, compared to 100% incidence and 30.0 attachments per plant in the susceptible control (**Table 2**).

**Table 2:** Percentage of susceptible plants (%S) and average number of *O. cumana* attachments in susceptible plants (Xs) in 12 sunflower hybrids resistant to *O. cumana* race F_GV_ and the susceptible control 63D82 evaluated with *O. cumana* population BR-28.

Hybrid	*n*	%S	Xs
Hybrid 1	6	100	11,7
Hybrid 2	9	89	3,0
Hybrid 3	10	100	12,8
Hybrid 5	9	56	2,6
Hybrid 6	10	100	5,8
Hybrid 7	10	100	4,8
Hybrid 8	10	100	2,9
Hybrid 9	10	70	1,4
Hybrid 10	5	100	11,6
Hybrid 11	10	100	5,4
Hybrid 12	10	100	5,5
Hybrid 13	9	100	7,0
63D82	10	100	30,0

### Interpopulation Relatedness

**FIGURE 1:**
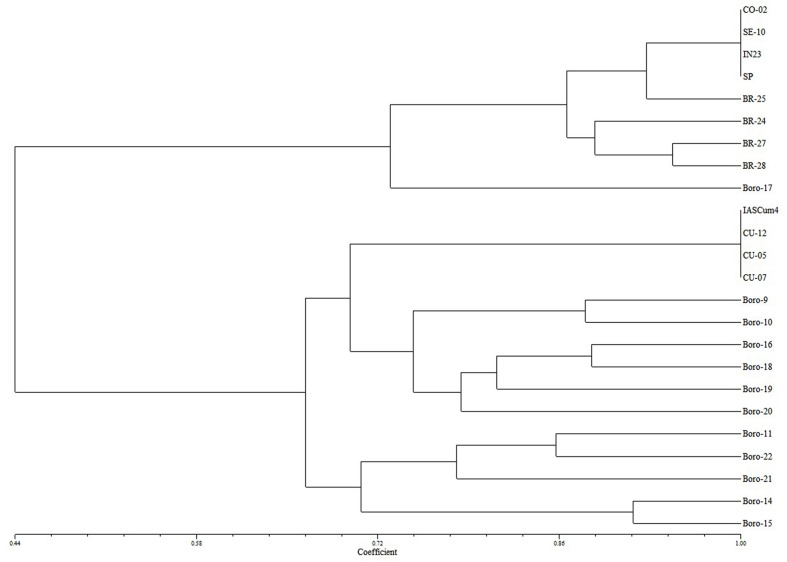
**Tree plot of the four populations under study (BR-24, BR-25, BR-27, BR-28) together with reference populations from the Guadalquivir Valley (CO-02, SE-10, EK-23, SP), Central Spain (IASCum-4, CU-05, CU-07, CU-12), Serbia (Boro-9), Romania (Boro-10), Israel (Boro-11), Turkey (Boro-14, Boro-15), and Bulgaria (Boro-16 to Boro-22)**.

Cluster analysis revealed that the four populations with increased virulence (BR-24, BR-25, BR-27, and BR-28) were genetically related to conventional populations of the Guadalquivir Valley (**Figure 1**). The analysis, with a high cophenetic correlation coefficient (*r* = 0.86, *P* < 0.01), resulted in two main clusters, one formed by the eight populations of the Guadalquivir Valley and one population of Bulgaria at a greater distance, and a second cluster including the populations from Cuenca together with all other populations from Israel, Serbia, Romania, Turkey, and Bulgaria.

### Genetic Diversity of the New Populations

**FIGURE 2:**
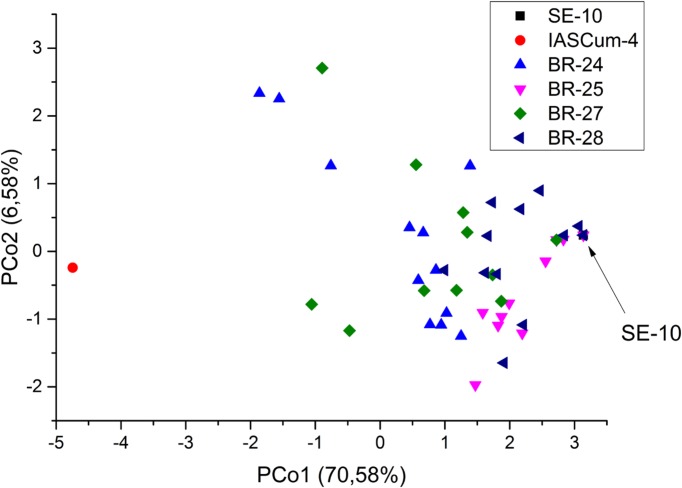
**Principal coordinates analysis of four *Orobanche cumana* populations (BR-24, BR-25, BR-27, BR-28) from the Guadalquivir Valley with increased virulence, and reference populations from the Guadalquivir Valley (SE-10) and Central Spain (IASCum-4)**.

**FIGURE 3:**
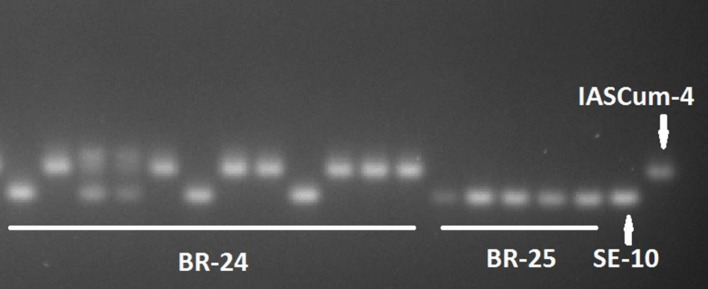
**Detail of an electrophoresis gel showing allelic variation at the Ocum-197 SSR locus in 12 single plants of *O. cumana* population BR-24, 5 single plants of population BR-25, and the reference populations from the Guadalquivir Valley (SE-10) and Central Spain (IASCum-4)**.

Compared with the population of the Guadalquivir Valley used as a reference (SE-10, race F), the four populations collected on race F_GV_ resistant hybrids showed considerably greater genetic diversity (**Figure 2**). The four populations showed greater values of all the genetic parameters evaluated, except the number of private alleles (**Table 3**). For example, the percentage of polymorphic loci ranged from 43.75 in BR-25 and BR-28 to 87.50 in BR-24, compared to zero in SE-10, while the average number of pairwise differences between individuals of a population ranged from 2.05 in BR-28 to 4.59 in BR-24, compared to zero in SE-10. The AMOVA confirmed the existence of intra-population diversity, which accounted for 75.6% of the total variance (**Table 4**). All polymorphic loci contained two alleles, the one found in populations of the Guadalquivir Valley and the one found in populations of Cuenca (**Figure 3**). No other alleles were present in the populations.

**Table 3:** Genetic diversity parameters of four *O. cumana* populations (BR-24, BR-25, BR-27, and BR-28) from the Guadalquivir Valley with increased virulence, and a reference population from the Guadalquivir Valley (SE-10).

Population	*P*	Na (±SE)	Na ≥5% (±SE)	Ne (±SE)	Npa (±SE)	H_0_ (±SE)	He (±SE)	I (±SE)	Pairwise differences
BR-24	87.50	1.88 ± 0.09	1.88 ± 0.09	1.65 ± 0.10	0.00 ± 0.00	0.19 ± 0.05	0.35 ± 0.05	0.51 ± 0.06	4.59
BR-25	43.75	1.44 ± 0.13	1.44 ± 0.13	1.26 ± 0.11	0.00 ± 0.00	0.05 ± 0.02	0.14 ± 0.05	0.21 ± 0.07	2.26
BR-27	68.75	1.69 ± 0.12	1.69 ± 0.12	1.49 ± 0.11	0.00 ± 0.00	0.05 ± 0.02	0.27 ± 0.05	0.39 ± 0.08	3.67
BR-28	43.75	1.44 ± 0.13	1.44 ± 0.13	1.25 ± 0.08	0.00 ± 0.00	0.05 ± 0.02	0.15 ± 0.05	0.23 ± 0.07	2.05
SE-10	0.00	1.00 ± 0.00	1.00 ± 0.00	1.00 ± 0.00	0.00 ± 0.00	0.00 ± 0.00	0.00 ± 0.00	0.00 ± 0.00	0.00

*P, percentage of polymorphic loci; Na, average observed allele number, Na >5%, number of different alleles with a frequency ≥5%; Ne, number of effective alleles; Npa, number of private alleles unique to a single population; Ho, observed heterozygosity; He, expected heterozygosity; I, Shannon’s diversity index; Pairwise differences: average number of pairwise differences between individuals within each population.*

**Table 4:** Analysis of molecular variance (AMOVA) in a set of four populations of *O. cumana* with increased virulence collected in the Guadalquivir Valley area (BR-24, BR-25, BR-27, and BR-28).

Source of variation	Sum of squares	variance components	% Variation	*P* value
Among populations	36.52	0.47	24.42	<0.01
Within populations	127.29	1.45	75.58	<0.01

## Discussion

Four populations of *O. cumana*, collected on race F_GV_ resistant hybrids in the Guadalquivir Valley area of Spain, were confirmed to have increased virulence as compared with conventional populations in that area. The four populations showed similar level of virulence and parasitized most of the race F_GV_ resistant genotypes evaluated, with level of incidence of 100% or close to it in most cases, with the exception of two lines (P96, DEB2) that were fully resistant and some hybrids that showed moderate or low level of incidence and degree of attack. The four populations from the Guadalquivir Valley clearly differed from race G populations from Eastern Europe for their inability to parasitize P96 line, while the reaction on race F resistant hybrids followed different patterns in the race G populations from Eastern Europe. Currently, race classification of *O. cumana* populations is based on sets of differential lines. However, there is no universal set of differential lines, as it is for example the case for downy mildew in this crop (Tourvieille de Labrouhe et al., 2000). Differential lines used for *O. cumana* are specific to individual seed companies or research groups, particularly for races above E (Fernández-Martínez et al., 2012). For populations of the Guadalquivir Valley, the line NR5 derived from the differential line P1380 (Vrânceanu et al., 1980) has been used as differential between races E and F_GV_, and line P-96 (Fernández-Martínez et al., 2004) as differential line with race F_GV_ resistance. The line P-96 was selected as differential line because it was completely resistant to race F_GV_ and to the race F predominant in Central Spain, and susceptible to the first reported race G, which was identified in the Edirne area of Turkey around 2000 (Kaya et al., 2004). P-96 line has been also reported to be susceptible to the race G present in Russia (Antonova et al., 2013) and the Black Sea area of Romania (Hladni et al., 2014). According to the results of the present study, it is also susceptible to race G populations from other areas. Therefore it becomes evident that the virulence of the new populations of the Guadalquivir Valley area is different to races G populations from Eastern Europe. We are accordingly naming the new populations as a local race G of the Guadalquivir Valley (G_GV_) to differentiate its level of virulence from the currently predominant race F_GV_ and from races G described for other areas such as Turkey (Kaya et al., 2004), Bulgaria (Shindrova and Penchev, 2012), Romania (Pacureanu-Joita et al., 2009), Russia (Antonova et al., 2013), China (Shi et al., 2015), Greece, and Ukraine (present study). This study also concluded that the inbred line P-96 is not valid as differential line between races F_GV_ and G_GV_, as it is resistant to both groups of populations. Research is ongoing to identify and release a public line to differentiate between both races.

In a previous study, Pineda-Martos et al. (2013) identified the presence of two genetically distant pools of *O. cumana* in Spain, one in the Guadalquivir Valley in southern Spain and another one in Cuenca province in Central Spain. The study identified extremely low interpopulation and intrapopulation genetic diversity within each gene pool, but also the existence of a few populations with greater genetic diversity that resulted from admixture between populations of both gene pools and subsequent hybridization. In the present study, the four populations classified as race G_GV_ were genetically related to the Guadalquivir Valley gene pool when compared with a set of populations of the two Spanish gene pools and from other areas, mainly Eastern Europe. This suggested that race G_GV_ populations have evolved *in situ* and do not correspond to new introductions from other areas.

Race G_GV_ populations were characterized by greater genetic diversity than the conventional populations of the Guadalquivir Valley (Pineda-Martos et al., 2013). The later study included 30 populations from the Guadalquivir Valley area, mainly belonging to races E and F_GV_, and even populations previous to race E. The low genetic diversity between populations and within populations was attributed to a founder effect, taking into account that *O. cumana* is not present in the wild in Spain (Pujadas-Salvà and Velasco, 2000). The high genetic similarity between race E and race F_GV_ populations suggested that race F_GV_ evolved *in situ* by punctual mutation from race E (Pineda-Martos et al., 2013). This was confirmed by Rodríguez-Ojeda et al. (2013b), who found segregation at a single locus in segregating populations from crosses between both races from the same gene pool. Conversely, genetic differentiation of race G_GV_ populations from the main gene pool of the Guadalquivir Valley suggests alternative mechanisms of race evolution. Genetic diversity parameters of the populations included in the present research are comparable to those reported by Pineda-Martos et al. (2013) in a set of populations with increased genetic diversity, with He values between 0.15 and 0.35 in this study compared with values between 0.12 and 0.49 in the previous one. Similarly to the study of Pineda-Martos et al. (2013), we have observed that the alleles present in polymorphic markers in the populations with increased virulence are those from the Guadalquivir Valley and Cuenca, with no new alleles being detected. Accordingly, we hypothesize that increased virulence in the four populations evaluated in the present research may have resulted from genetic recombination between avirulence genes present in both gene pools. Molinero-Ruiz et al. (2014) reported differences for virulence between race F populations from the Guadalquivir Valley and Cuenca province. Admixture between populations, hybridization and recombination of avirulence genes has been identified as an important mechanism to create increasing virulent races in sunflower downy mildew (Ahmed et al., 2012). In *Orobanche* spp., the occurrence of gene flow between populations has been previously documented in *O. minor* (Thorogood et al., 2009) and *O. cumana* (Pineda-Martos et al., 2014a).

The previous study of Pineda-Martos et al. (2013) included two populations with increased genetic diversity from the Guadalquivir Valley area, SE-02 and SE-07. The former was collected in 1991 and classified as race below E while the latter was collected in 1999 and classified as race E. The presence of alleles from the gene pool of Cuenca province was documented in both populations, although in that study greater genetic diversity was not associated with increased virulence. The reason for this could be that both gene pools did not differ for virulence alleles before the appearance of race F, although this has to be confirmed. The different situation in the populations collected in 2014 suggests that both gene pools may differ for race F virulence alleles and that their genetic recombination may be considered as a plausible mechanism underlying the recent appearance of the new race G_GV_.

## Author Contributions

LV, JF-M and AM-S conceived the work and planned the experiments. AM-S, LV, and JF-M conducted the phenotypic evaluations. BP-V coordinated the molecular markers research and conducted statistical analyses. JM conducted molecular markers analyses. AM-S and LV wrote the draft of the manuscript. All authors read it critically and contributed to the discussion.

## Conflict of Interest Statement

The manuscript is the result of public-private research collaboration, between the company Dupont Pioneer and the Institute for Sustainable Agriculture (IAS), which belongs to the Spanish National Scientific Research Council. Research conducted at the company has been funded by the own company, whereas research conducted at IAS has been funded by research project AGL2014-53886-P of the Spanish Ministry for Economy and Innovation, with involvement of EU FEDER Funds. Collaboration has been based on interchange of ideas and plant material, which led to the design of experiments of interest for both parties and the preparation of the present manuscript to inform the scientific community on the results of our experiments. For the sake of the experiments, sunflower commercial hybrids from seed companies other than Dupont Pioneer were included in the research. Both Dupont Pioneer and IAS consider they would have a conflict of interest if the names of the commercial hybrids and their corresponding companies were reported together with their performance under artificial infestation with an alleged new race of sunflower broomrape that is being reported for the first time in this manuscript. Since the objective of the research was not to conduct a survey on resistance of commercial hybrids to a new race, but just to ensure that the new populations under study can be considered as a new race, the hybrids were not selected following the criterion of representativeness, and some seed companies may be overrepresented and others not included in the study, which might result in an unbalanced impact on the commercial interests of seed companies. For this reason, the names of the hybrids and their corresponding companies have been omitted in the manuscript.
